# Metabolomics reveals metabolites associated with hair follicle cycle in cashmere goats

**DOI:** 10.1186/s12917-024-04057-0

**Published:** 2024-05-17

**Authors:** Shengchao Ma, Wenzhi Cao, Xiaolin Ma, Xiaofang Ye, Chongkai Qin, Bin Li, Wenna Liu, Qingwei Lu, Cuiling Wu, Xuefeng Fu

**Affiliations:** 1 Key Laboratory of Special Environments Biodiversity Application and Regulation in Xinjiang, College of Life Sciences, Xinjiang Normal University, Xinjiang, Urumqi, 830017 China; 2 Xinjiang Key Laboratory of Special Species Conservation and Regulatory Biology, College of Life Sciences, Xinjiang Normal University, Xinjiang, Urumqi, 830017 China; 3 Key Laboratory of Genetics Breeding and Reproduction of Xinjiang Wool-sheep Cashmere-goat (XJYS1105), Institute of Animal Science, Xinjiang Academy of Animal Sciences, Xinjiang, Urumqi, 830011 China; 4 Xinjiang Aksu Prefecture Animal Husbandry Technology Extension Center, Xinjiang, Aksu, 843000 China

**Keywords:** Cashmere goat, Hair follicle cycle, Metabolome of skin tissue, Relationships, Influencing factors

## Abstract

**Background:**

The hair follicle is a skin accessory organ that regulates hair development, and its activity varies on a regular basis. However, the significance of metabolites in the hair follicle cycle has long been unknown.

**Results:**

Targeted metabolomics was used in this investigation to reveal the expression patterns of 1903 metabolites in cashmere goat skin during anagen to telogen. A statistical analysis was used to investigate the potential associations between metabolites and the hair follicle cycle. The findings revealed clear changes in the expression patterns of metabolites at various phases and in various feeding models. The majority of metabolites (primarily amino acids, nucleotides, their metabolites, and lipids) showed downregulated expression from anagen (An) to telogen (Tn), which was associated with gene expression, protein synthesis and transport, and cell structure, which reflected, to some extent, that the cells associated with hair follicle development are active in An and apoptotic in An–Tn. It is worth mentioning that the expression of vitamin D3 and 3,3’,5-triiodo-L-thyronine decreased and then increased, which may be related to the shorter and longer duration of outdoor light, which may stimulate the hair follicle to transition from An to catagen (Cn). In the comparison of different hair follicle development stages (An, Cn, and Tn) or feeding modes (grazing and barn feeding), Kyoto Encyclopedia of Genes and Genomes (KEGG) enrichment analysis revealed that common differentially expressed metabolites (DEMs) (2’-deoxyadenosine, L-valine, 2’-deoxyuridine, riboflavin, cytidine, deoxyguanosine, L-tryptophan, and guanosine-5’-monophosphate) were enriched in ABC transporters. This finding suggested that this pathway may be involved in the hair follicle cycle. Among these DEMs, riboflavin is absorbed from food, and the expression of riboflavin and sugars (D-glucose and glycogen) in skin tissue under grazing was greater and lower than that during barn feeding, respectively, suggesting that eating patterns may also alter the hair follicle cycle.

**Conclusions:**

The expression patterns of metabolites such as sugars, lipids, amino acids, and nucleotides in skin tissue affect hair follicle growth, in which 2’-deoxyadenosine, L-valine, 2’-deoxyuridine, riboflavin, cytidine, deoxyguanosine, L-tryptophan, and guanosine-5’-monophosphate may regulate the hair follicle cycle by participating in ABC transporters. Feeding practices may regulate hair follicle cycles by influencing the amount of hormones and vitamins expressed in the skin of cashmere goats.

**Supplementary Information:**

The online version contains supplementary material available at 10.1186/s12917-024-04057-0.

## Background

One of the most prominent physical characteristics of mammals, fur, hair, wool, or cashmere, has a variety of purposes, including regulating body temperature and providing physical protection, among others, and is also crucial for animal social behavior [1, 2]. Moreover, the hair, wool, or cashmere of certain animals has significant economic significance since it may be utilized as a textile material. For example, cashmere from goats (Capra hircus) is a high-quality textile material with a glossy, silky, and elastic texture, but its production has difficulty meeting market demand. Therefore, improving the quality and yield of cashmere is currently an important task in cashmere goat breeding.

As the control center of cashmere growth [3–5], multiple studies have established the developmental pattern of hair follicles, which are cyclic in growth and may be loosely divided into An, Cn, and Tn, in cashmere goats [6–8]. Cashmere goats exhibited sequential changes in three stages with different seasons. In An, new hair grows in the hair follicle, and the hair bulb proliferates, differentiates, and grows upward. Cn is the shortest of these three stages, during which cell division in the hair matrix ceases and the lower half of the hair follicle recedes and shrinks. Finally, in Tn, hair growth slows, and energy is conserved for the following hair follicle cycle. The study of the regulatory mechanism and influencing factors of the hair follicle cycle is important for enhancing goat cashmere quality and yield.

With further research, many genes, molecular markers, and signaling pathways related to the hair follicle cycle and traits such as wool fiber diameter, density, and yield, such as *KRTs*, *KRTAPs* [9], *BMPs* [10–12], *IGFs* [13–15], *EGFs* [16, 17], *EDAR* [18], *FA2H* [19, 20], Wnt/β-catenin [21], Sonic Hedgehog (*shh*) [22, 23], and *Notch* [24], have been identified by previous studies at the genomic or transcriptomic level for the genetic improvement of cashmere traits in different goat breeds.

However, the importance of particular metabolites (e.g., amino acids and their metabolites, nucleotides and their metabolites, lipids, vitamins, and coenzymes) in the hair follicle cycle cannot be overlooked. They can be engaged in the regulation of animals’ metabolic processes, providing substrates and energy for these metabolic activities, or they can be employed directly as components of cells and tissues. For instance, Ohnemus et al. reported that estrogen can induce hair follicles to enter Cn and maintain hair follicles in Tn [25]. According to Craven’s research, prolactin can cause the death of hair parent keratinocytes and prevent the growth of hair follicle keratinocytes, which helps hair follicles enter Cn earlier [26]. Moreover, vitamins also have an impact on hair development [27]. In addition, external factors may influence animal development and metabolic activity by directly influencing the quantities of certain metabolites in the body [28, 29]. For example, light regulates the growth of hair by affecting the synthesis of melatonin in skin tissue [30, 31]. To date, the specific role played by various metabolites and the intrinsic relationship between external factors and metabolite changes are not fully understood. As a result, it is critical to investigate the expression of metabolites in cashmere goat hair follicles/skin tissues in all three stages, and this is an important part of research on the regulatory mechanism of hair growth.

Metabolomics, an emerging omics technique following genomics, transcriptomics, and proteomics, provides the technical foundation for a thorough understanding of the roles of various metabolites in the hair follicle cycle. Metabolomics quantifies and characterizes all small molecule (molecular weight less than 1000 Da) endogenous metabolites downstream of gene regulatory and protein action networks. Metabolomics provides a comprehensive perspective of the changes in the types and amounts of numerous substances in an organism during metabolism, allowing for an accurate and direct portrayal of an animal’s physiological condition and phenotype [32]. In addition, metabolomics can help us explore the metabolite interactions underlying the aforementioned variations. Metabolomics has been widely used in human or animal disease [33, 34] and nutrition research [35, 36] in recent years, with a focus on biomarker metabolites in specific disease conditions, the relationship between disease and diet, and the effects of certain food components or additives on animal development. However, there have been no studies of metabolite expression patterns in animal hair follicles or skin tissues from An to Tn.

In this study, we used the Jiangnan cashmere goat, an important goat breed in Xinjiang, as the research object and collected skin tissues from Jiangnan cashmere goats during the An, Cn, and Tn under grazing and barn feeding. For the first time, the temporal metabolite expression profiles of skin tissues during the three hair growth stages were generated using extensive high-throughput targeted metabolomics. Then, statistical analyses were used to screen the important DEMs at various stages and identify the pathways associated with the hair follicle cycle. Finally, the potential relationships between various metabolites and the hair follicle cycle were revealed. This study provides a theoretical foundation for future research on the regulatory mechanisms of the hair follicle cycle, as well as the nutritional management and breeding of cashmere goats.

## Results

### PCA and correlation analysis of 36 metabolome samples

**Fig. 1:**
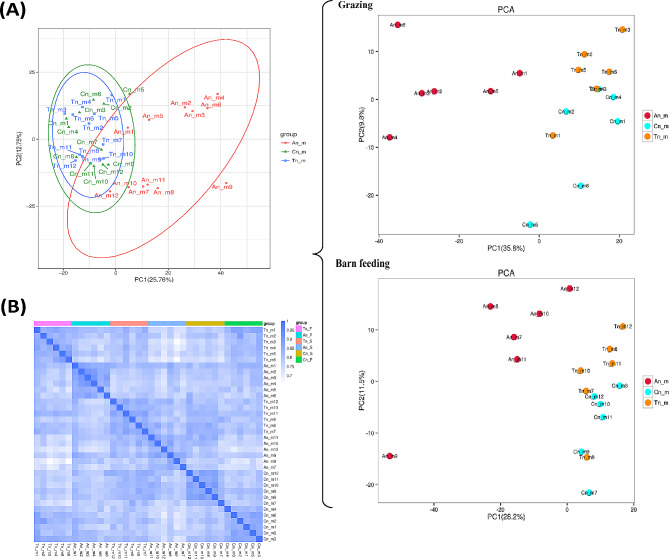
PCA and repeat correlation analysis of 36 metabolome samples. (**A**) All samples were first subjected to PCA. Then, depending on the feeding model, all samples were separated into grazing (F) and barn feeding (S) groups, and PCA (three PCA scatter plots in braces) of samples under different feeding modes was performed. Samples 1–6 (An_m1-An_m6, Cn_m1-Cn_m6, and Tn_m1-Tn_m6) were from Jiangnan cashmere goats raised under grazing and samples 7–12 (An_m7-An_m12, Cn_m7-Cn_m12, and Tn_m7-Tn_m12) were from Jiangnan cashmere goats raised under barn feeding. (**B**) Correlation heatmap between samples

PCA of 36 metabolome samples showed that the metabolite expression patterns of samples from the An group (including 12 samples: An_m1 to An_m12) were obviously different from those of samples from the Cn (including 12 samples: Cn_m1 to Cn_m12) and Tn (including 12 samples: Tn_m1 to Tn_m12) groups and were similar between samples from the Cn and Tn groups (Fig. 1A). Under grazing, the distribution of samples in the An (including 6 samples: An_m1 to An_m6), Cn (including 6 samples: Cn_m1 to Cn_m6), and Tn (including 6 samples: Tn_m1 to Tn_m6) groups, respectively, was comparable to that (including 18 samples: An group: An_m7 to An_m12; Cn group: Cn_m7 to Cn_m12; and Tn group: Tn_m7 to Tn_m12) under barn feeding (Fig. 1A). Intersample correlation analysis revealed a strong correlation between two replicate samples within the same grouping and some correlation between samples from the Cn and Tn groups (Fig. 1B).

### Overview of metabolites in 36 skin tissues

In 36 skin tissues, a total of 1903 metabolites were detected. Among these metabolites, amino acids and their metabolites, fatty acyls, organic acids and their derivatives, and nucleotides and their metabolites were predominant, with the largest kinds of amino acids and their metabolites detected (358 in total), followed by organic acid and its derivatives and nucleotides and their metabolites (138 and 107, respectively). The results further showed that among the 358 amino acids and their metabolites detected, 209 were small peptides, 106 were amino acid derivatives, and 42 were amino acids (Table 1). In addition, 19 hormones and 12 co-enzymes or vitamins were included in the detected metabolites, with the hormones mainly including progesterone, thyroxine, norepinephrine, and epinephrine, and the co-enzymes or vitamins mainly including B vitamins and D vitamins.

**Table 1:** Statistics of metabolite composition types in skin tissue

Class I	Class II	kinds
Amino acid and its metabolites	Small Peptide	209
	Amino acid derivatives	106
	Amino acids	42
	Amino acid and Its metabolites	1
Benzene and substituted derivatives	Benzene and substituted derivatives	37
	Phenolic acids	15
	Phenolics	9
Alcohol and amines	Amines	22
	Polyamines	12
	Alcohols	4
Bile acids	Bile acids	18
CoEnzyme and vitamins	CoEnzyme and vitamins	12
Glyceryl phosphatide (GP)	Lyso-phosphatidylcholine (LPC)	37
	Lysophosphatidyl ethanolamine (LPE)	23
	Lysophosphatidic acid (LPA)	3
	phosphatidyl inositol (PI)	1
	Lysophosphatidyl choline	1
	Phosphatidyl ethanolamine (PE)	1
	Phosphatidylcholine (PC)	1
Glycerolipid (GL)	Triglyceride (TG)	1
Nucleotide and its metabolites	Nucleotide and its metabolites	107
Hormones and hormone related compounds	Hormones and hormone related compounds	19
Others	Others	4
Sphingolipid	Sphingomyelin	3
	Sphingosinol	1
Tryptamines, cholines, pigments	Cholines	2
	Tryptamines	2
Carbohydrates and Its metabolites	Sugars	27
	Phosphate sugars	11
	Sugar acids	8
	Carbohydrates and Its metabolites	6
	Sugar alcohols	5
	Sugar derivatives	3
Organic acid and Its derivatives	Organic acid And Its derivatives	123
	Sulfonic acids	8
	Phosphoric acids	7
Heterocyclic compounds	Heterocyclic compounds	26
	Pteridines and derivatives	12
	Indole and Its derivatives	7
	Pyridine and pyridine derivatives	1
Fatty acyl (FA)	Acylcarnitine	50
	Oxidized lipids	81
	Free fatty acid (FFA)	23
	Others	2

### Analysis of the expression trends of metabolites from an to Tn

#### The overall trend of metabolite expression in skin tissue from 12 cashmere goats

**Fig. 2:**
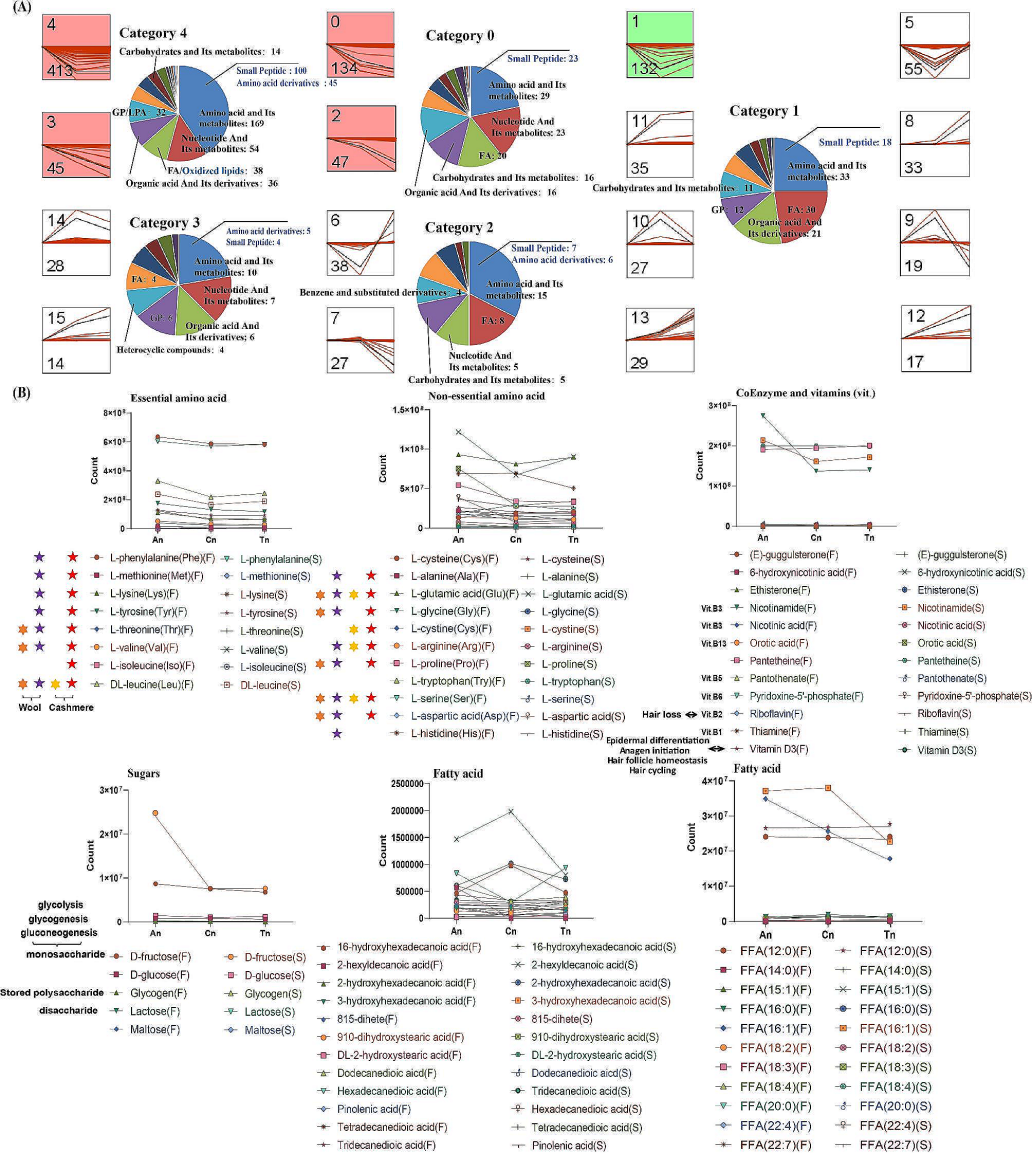
Expression trend analysis of metabolites from An to Tn. (**A**) Line graphs reflecting the expression trends of metabolites in different categories. The numbers in the upper left corner and the numbers in the lower left corner of the line graph represent the category number and the kinds of metabolites contained in the category, respectively. The pie charts show the percentages of different kinds of metabolites. (**B**) During An to Tn, the expression trends of amino acids, carbohydrates, vitamins, and fatty acids were studied using various feeding models. The amino acids identified with five-pointed purple stars are wool keratin components, whereas the orange six-pointed stars represent amino acids that are highly expressed in wool [37, 38]. The amino acids identified with five-pointed red stars are cashmere keratin components, whereas the yellow six-pointed stars represent amino acids that are highly expressed in cashmere [39, 40]

Expression trend analysis was performed using the expression of all metabolites in 36 skin tissues. The results showed that all metabolites were classified into 16 categories, with category 4, category 3, category 2, category 0, and category 1 as significant categories, and these 4 categories including the majority of metabolites detected (Fig. 2A and Table S1). Meanwhile, metabolites in category 4, category 3, category 2, and category 0 showing overall downregulated expression trends (Fig. 2A and Table S1). Notably, category 4 contained 54 nucleotides and their metabolites (including cytosine, guanine, hypoxanthine, ADP ribose, guanosine, guanosine-5’-monophosphate, 2’-deoxyadenosine, etc.), 18 amino acids (including L-lysine, L-arginine, L-asparagine anhydrous, L-proline, L-glycine, L-alanine, L-valine, L-tyrosine, L-phenylalanine, L-tryptophan, etc.), 33 fatty acyls (including arachidonic acid (AA), etc.), nicotinamide, orotic acid, thiamine, D-fructose, 1,6-Di-O-phosphono-D-fructose, fumaric acid, orotic acid, oxaloacetic acid, acetylcholine, and acrylamide (Table S1).

In addition, category 0 included D-glucose, uric acid, nicotinic acid, pantothenate, riboflavin, D-malic acid, uridine 5’-diphosphate, uric acid, adenine, and thymine; category 2 included L-histidine, L-threonine, and UDP-glucose; category 3 included Dl-glyceraldehyde 3-phosphate, uridine, xanthine, and P-coumaric acid; and category 1 included lactose, maltose, prostaglandin D_2_ (PGD_2_), L-cystine, L-glutamic acid, and citric acid (Table S1).

#### Metabolite expression trends in skin tissues under different feeding modes

The 36 metabolome samples were divided into S and F groups to study the expression of metabolites under different feeding modes (Fig. 2B and Table S2). The more detailed results showed that the expression of most essential and nonessential amino acids (including L-phenylalanine, L-methionine, L-lysine, L-tyrosine, L-valine, and L-alanine) was still obviously downregulated or close to normal (including L-threonine, DL-leucine, L-cysteine, L-glycine, L-cysteine, L-arginine, L-proline, L-tryptophan, L-aspartic acid, and L-histidine) under both barn feeding and grazing, but L-glutamic acid was obviously downregulated and then slightly upregulated under barn feeding, and L-serine was slightly upregulated and then downregulated under grazing (Fig. 2B and Table S2).

Moreover, the expression of some sugars (e.g., D-fructose, L-glucose, and glycogen) and vitamins (e.g., nicotinamide, orotic acid, nicotinic acid, thiamine, pantothenate, and riboflavin) followed a trend similar to that of the amino acid expression under both barn feeding and grazing, and the decreasing trend of D-fructose and nicotinamide was obvious under barn feeding and grazing, respectively, from An to Cn. The expression of lactose and maltose decreased slightly and then slightly increased under barn feeding and grazing (Fig. 2B and Table S2).

Finally, the expression trend of most fatty acids was more complicated. The expression of 2-hydroxyhexadecanoic acid and 16-hydroxyhexadecanoic acid was obviously downregulated and then upregulated under both barn feeding and grazing conditions (Fig. 2B and Table S2). The expression of 2-hexyldecanoic acid was obviously upregulated and then downregulated under barn feeding, and the expression of hexadecanedioic acid was obviously downregulated and then upregulated under grazing (Fig. 2B and Table S2).

### Analysis of DEMs

#### Comparison between the three hair follicle development stages

**Fig. 3:**
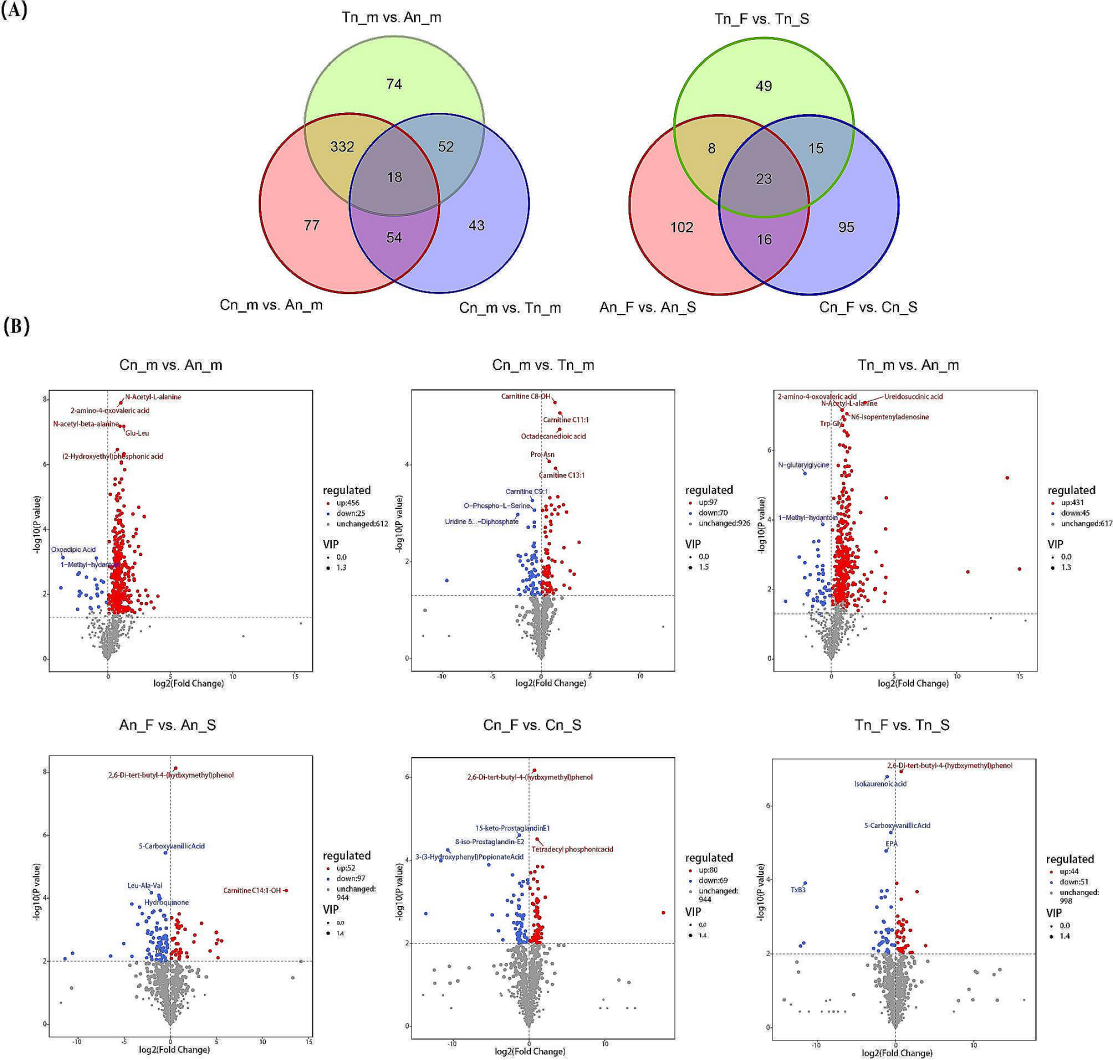
Differential expression analysis of skin metabolomes at different stages or under different feeding modes. (**A**) Venn diagram of the DEMs in the six comparison groups. (**B**) Volcano diagram of the DEMs in the six comparison groups

In An vs. Cn (An_m vs. Cn_m), a total of 481 DEMs (including L-valine, L-alanine, L-glutamic acid, L-glutamine, L-aspartic acid, L-lysine, L-phenylalanine, L-tryptophan, and L-methionine) were identified (Fig. 3A and B, Table S3). A total of 167 DEMs (including L-tyrosine, L-alanine, L-valine, L-glutamine, L-phenylalanine, L-tryptophan, L-lysine, L-aspartic acid, and L-methionine) were screened in Tn vs. Cn (Tn_m vs. Cn_m) (Fig. 3A and B, Table S4). A total of 474 DEMs were identified in An vs. Tn (An_m vs. Tn_m) (Fig. 3A and B, Table S5). Venn diagram analysis (Fig. 3A) further revealed 350 common DEMs in An vs. Cn and An vs. Tn, 72 common DEMs in An vs. Cn and Tn vs. Cn, 70 common DEMs in An vs. Tn and Tn vs. Cn, and 18 common DEMs (carnitine C14:2, carnitine C18:2, carnitine C8-OH, LPC(0:0/14:0), Dl-glyceraldehyde 3-phosphate, guanosine-5’-monophosphate, etc.) in these three comparison groups.

#### Comparison between different feeding models

In An_F vs. An_S, a total of 149 DEMs (including L-tryptophan, L-cystine, and L-cysteine) were screened (Fig. 3A and B, Table S6). In Cn_F vs. Cn_S, 149 DEMs (the amino acids included L-arginine) were also screened (Fig. 3A and B, Table S7). Finally, a total of 95 DEMs (whose amino acids included L-serine) were screened in Tn_F vs. Tn_S (Fig. 3A and B, Table S8). Venn diagram analysis (Fig. 3A) revealed 39 common DEMs in An_F vs. An_S and Cn_F vs. Cn_S, 31 common DEMs in An_F vs. An_S and Tn_F vs. Tn_S, and 38 common DEMs in Cn_F vs. Cn_S and Tn_F vs. Tn_S. Moreover, 23 common DEMs (EPA, AA, FFA (22:4), 2’-deoxyadenosine, FFA(18:2), 5’-deoxyadenosine, Arg-Ser, etc.) were screened in these three comparison groups.

### KEGG enrichment analysis of DEMs

#### KEGG enrichment analysis of the DEMs of an vs. cn, tn vs. cn and an vs. tn

KEGG enrichment analysis of the DEMs in the 3 comparison groups revealed that the DEMs in An vs. Cn were enriched mainly in ABC transporters (including 2’-deoxyadenosine, L-valine, 2’-deoxyuridine, riboflavin, deoxyguanosine, D-trehalose, and cytidine), purine metabolism (including deoxyguanosine, guanine, guanosine-5’-monophosphate, 2’-deoxyadenosine, and allantoic acid), biosynthesis of alkaloids derived from the shikimate pathway, biosynthesis of plant secondary metabolites, and pyrimidine metabolism (including tryptamine, guanosine-5’-monophosphate, L-valine, oxaloacetic acid, and L-tryptophan) (Table S9).

The DEMs in An vs. Tn were mainly enriched in the biosynthesis of alkaloids derived from the shikimate pathway, pyrimidine metabolism (including uridine, thymine, cytidine, cytosine, thymidine, 3-ureidopropionate and 2’-deoxyuridine), purine metabolism (including uridine, thymine, cytidine, cytosine, thymidine, 3-ureidopropionate and 2’-deoxyuridine), ABC transporters (including riboflavin, spermidine, uridine, cytidine, 2’-deoxyuridine, deoxyguanosine, 2’-deoxyadenosine and L-valine) and biosynthesis of plant secondary metabolites (Table S10).

In addition, the DEMs in Tn vs. Cn were mainly enriched in the cAMP signaling pathway (including (R)-3-hydroxybutanoic acid and prostaglandin E2), ABC transporters (including aminolevulinic acid and D-trehalose), biosynthesis of plant secondary metabolites, glycine, serine and threonine metabolism (ko00260) (including aminolevulinic acid and 1,3-diaminopropane), purine metabolism (including guanosine-5’-monophosphate and 5-aminoimidazole ribonucleotide), neuroactive ligand (including guanosine-5’-monophosphate and 5-aminoimidazole ribonucleotide), neuroactive ligand‒receptor interaction (including cortisol and prostaglandin E2), butanoate metabolism, beta-alanine metabolism, pathways in cancer (including prostaglandin E2 and cortisol), cutin, suberin and wax biosynthesis (including 9,10-dihydroxystearic acid and 16-hydroxyhexadecanoic acid), arginine and proline metabolism (including creatinine, 1-methyl-hydantoin and 1,3-diaminopropane) (Table S11).

#### KEGG enrichment analysis of the DEMs of An_F vs. An_S, Cn_F vs. Cn_S and Tn_F vs. Tn_S

The DEMs in An_F vs. An_S were mainly enriched in bile secretion (including chenodeoxycholic acid, acetaminophen + estrone 3-sulfate, and spermidine), ABC transporter (including spermidine, 2’-deoxyadenosine, and L-cystine) and arginine and proline metabolism (including 1-methyl-hydantoin, creatine, and spermidine) pathways (Table S12). The DEMs in Cn_F vs. Cn_S were predominantly enriched in ABC transporters (including uridine, cytidine, 2’-deoxyadenosine, riboflavin, and deoxyguanosine) and pyrimidine metabolism (including cytidine, thymidine, cytosine, and uridine) (Table S13). The DEMs in the Tn_F vs. Tn_S comparison were enriched mainly in the glycine, serine, and threonine metabolism pathways (including the L-homoserine, L-serine and L-allothreonine pathways) (Table S14).

### Receiver operating characteristic (ROC) curve analysis

In An vs. Cn, Tn vs. Cn and An vs. Tn, there were 474, 164, and 469 DEMs with AUCs ≥ 0.7, respectively (Tables S3, S4 and S5). Moreover, we further analyzed the AUC values of the DEMs under different feeding models. There were 104 DEMs with AUCs > 0.95 in both An_F vs. An_S and Cn_F vs. Cn_S (Tables S6 and S7). Sixty-seven DEMs with AUCs > 0.95 were identified in the Tn_F vs. Tn_S comparison (Table S8).

### Metabolite expression patterns associated with animal physiological activity, growth, and development

Based on the results of the expression trend analysis, DEM analysis, and ROC analysis, we focused on the expression patterns of related metabolites in some basal metabolic pathways. These metabolites are strongly associated with animal physiological functions, such as genetic material expression and replication; energy metabolism; sugar, fat, and protein production and breakdown; and anti-stress.

#### Expression patterns of metabolites in the basal metabolic pathway

**Fig. 4:**
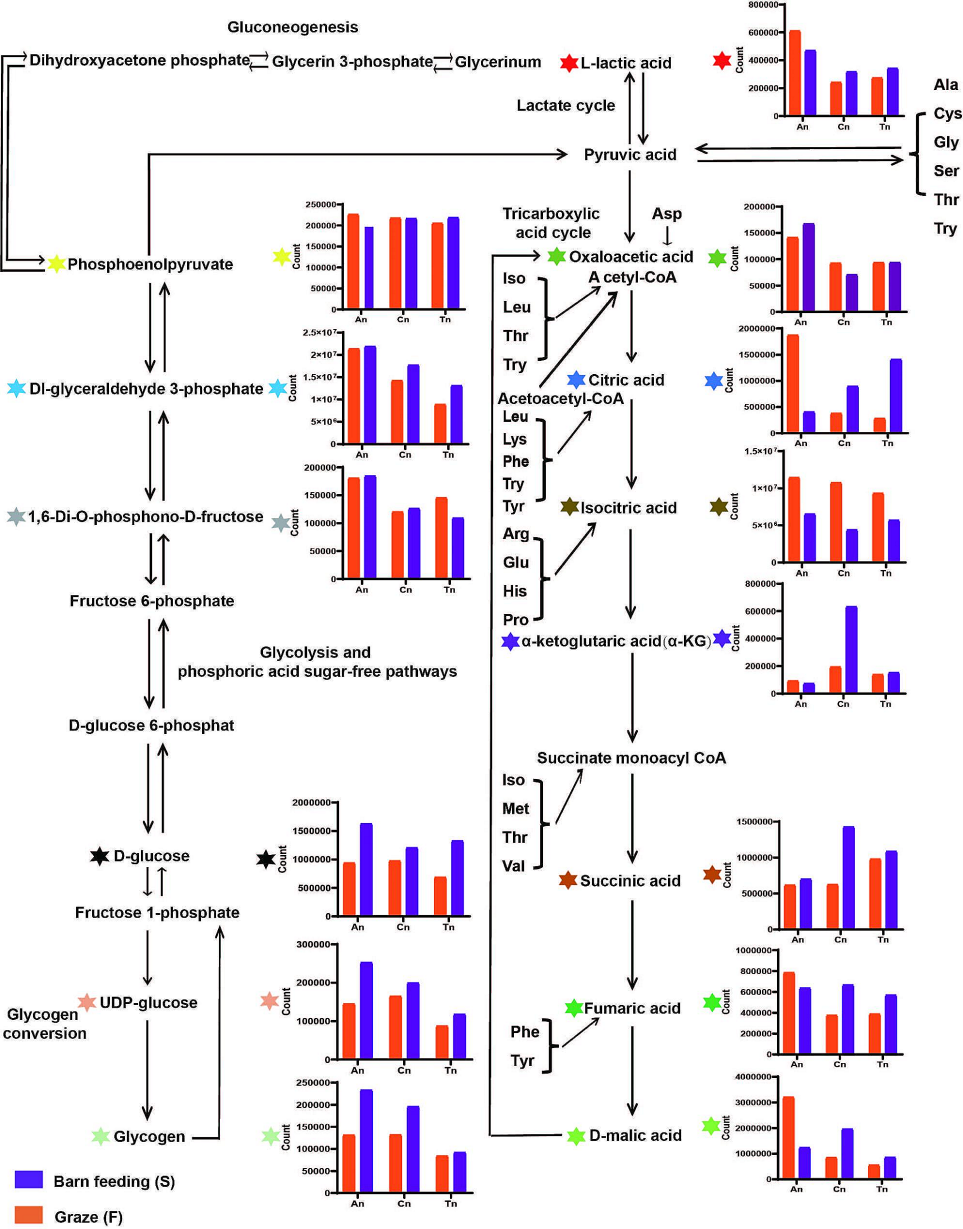
Expression patterns of metabolites associated with gluconeogenic pathways (glycogen formation and catabolism, phosphate-free pathway, glycolysis, lactate formation and catabolism, gluconeogenic pathway, and tricarboxylic acid cycle). Some metabolites and their corresponding expression columns are labeled with colored six-point stars

For glycogen conversion, the overall expression of glycogen (*P* < 0.01, variable importance in projection (VIP): 2.48, and area under the curve (AUC): 0.91 for Cn vs. Tn; *P* < 0.01, VIP: 1.20, and AUC: 0.85 for Tn vs. An) and UDP-glucose (*P* < 0.05, VIP: 1.83, and AUC: 0.80 for Cn vs. Tn; *P* < 0.01, VIP: 1.36, and AUC: 0.90 for Tn vs. An) decreased from An to Tn (Figs. 2B and 4), and the expression of D-glucose and glycogen was lower in the F group than in the S group, as was that of UDP-glucose (Fig. 4).

In the glycolysis or phosphoric acid sugar-free pathway, the expression patterns of related metabolites differed: the expression of DL-glyceraldehyde 3-phosphate (*P* < 0.05, VIP: 1.16, and AUC: 0.8125 in Cn vs. An; *P* < 0.01, VIP: 1.90, and AUC: 0.80 in Cn vs. Tn; *P* < 0.01, VIP: 1.65 and AUC: 0.95 in An vs. Tn) and 1,6-Di-O-phosphono-D-fructose showed a downward trend from An to Tn (Fig. 4). Moreover, the differences in the expression of phosphoenolpyruvate, Dl-glyceraldehyde 3-phosphate, and 1,6-Di-O-phosphono-D-fructose between the F and S groups were not significant (Fig. 3). Further analysis revealed that the expression of L-lactic acid (*P* < 0.01, VIP: 1.90, and AUC: 0.94 in Cn vs. An; *P* < 0.01, VIP: 1.56, and AUC: 0.95 in An vs. Tn) decreased from An to Tn, and the expression of L-lactic acid in An was greater in the F group than in the S group, while the opposite was true for Cn and Tn (Fig. 4).

Finally, in the tricarboxylic acid cycle (TCA) (Fig. 4), some intermediates exhibited similar expression patterns. The expression of oxaloacetic acid (*P* < 0.01, VIP: 1.5, and AUC: 0.92 in Cn vs. An; *P* < 0.01, VIP: 1.24 and AUC: 0.84 in An vs. Tn), citric acid, isocitric acid, and fumaric acid (log_2_FC: 0.57, *P* < 0.01, VIP: 1.20, and AUC: 0.83 in An vs. Tn), and D-malic acid (log_2_FC: -0.99, *P* < 0.05, VIP: 1.74, and AUC: 0.78 in Cn vs. Tn) was downregulated under grazing from An to Tn, and α-ketoglutaric acid (*P* < 0.05, VIP: 1.19, and AUC: 0.92 in Cn vs. An; *P* < 0.05, VIP: 1.66, and AUC: 0.81 in Cn vs. Tn), succinic acid, fumaric acid, and D-malic acid (*P* < 0.05, VIP: 1.74, and AUC: 0.78 in Cn vs. Tn) showed an up- and then downregulated expression trend under Furthermore, when the expression differences of metabolites in different feeding patterns were compared, succinic acid (log_2_FC: 1.19, *P* < 0.01, VIP: 1.64 and AUC: 0.94) and D-malic acid (log_2_FC: 1.21, *P* < 0.01, VIP: 1.54, and AUC: 0.89) were significantly different in the Cn group, while fumaric acid (log_2_FC: 0.55, *P* < 0.01, VIP: 1.56, and AUC: 0.92) was significantly different in the Tn group.

**Fig. 5:**
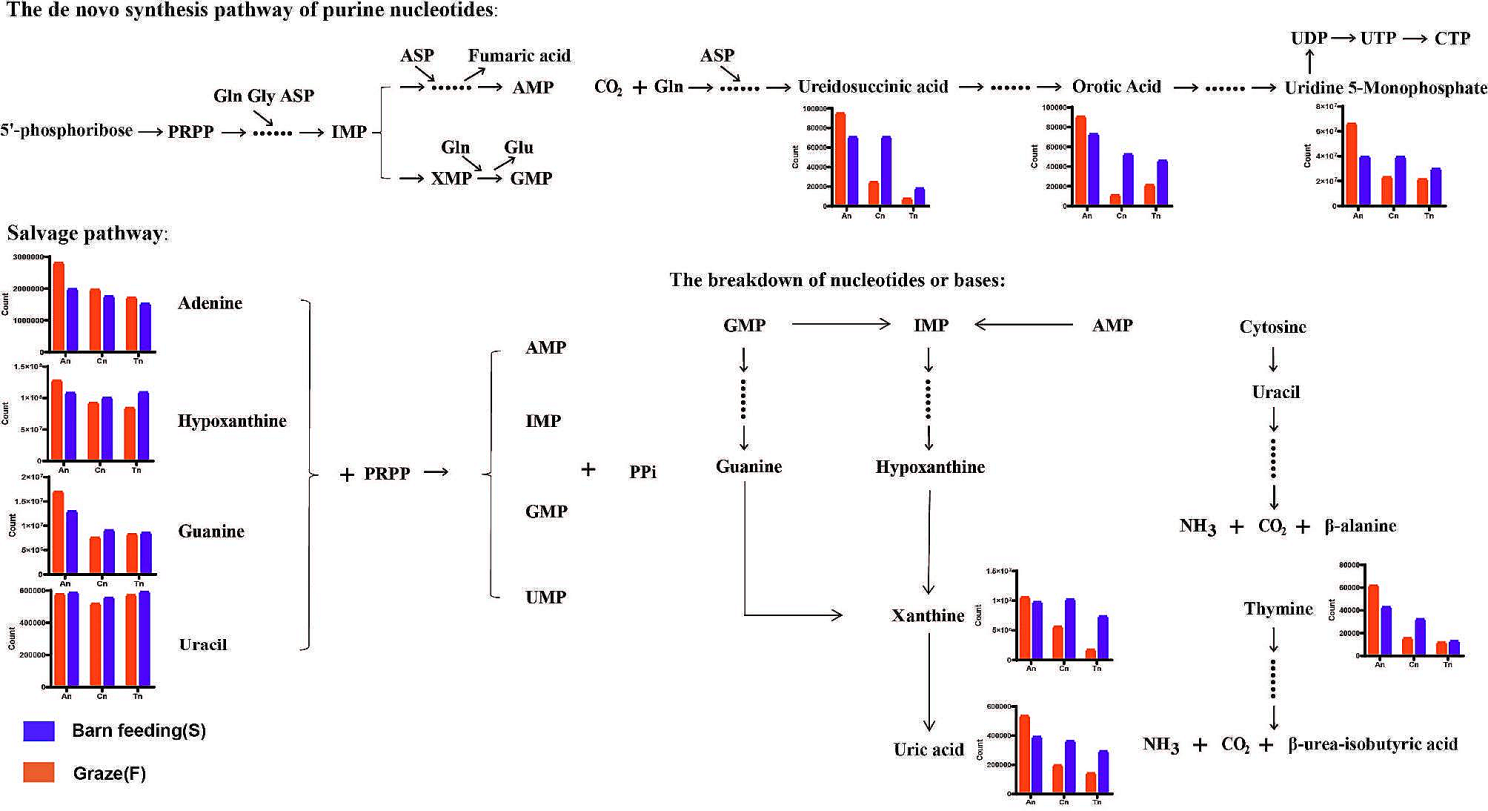
Expression patterns of metabolites associated with nucleotide and base synthesis and decomposition (de novo synthesis pathways, remediation pathways, and degradation)

Subsequently, we analyzed the expression patterns of metabolites involved in nucleotide and base biosynthesis and catabolism (Fig. 5). The expression of adenine, hypoxanthine, guaninic acid, and uracil (*P* < 0.05, VIP: 1.59, and AUC: 0.71 in Cn vs. Tn) in the salvage pathway showed a downward trend, but there was no significant difference in the expression of these metabolites under the different feeding modes.

Moreover, for the de novo synthesis of pyrimidine nucleotides (Fig. 5), the downregulated expression of ureidosuccinic acid, orotic acid, and uridine 5-monophosphate was more apparent in the F group than in the S group. This phenomenon was also reflected in the xanthine and uric acid degradation of purine nucleotides. Thymine had the most severe downregulated expression trend among the purine bases and pyrimidine bases.

#### Expression patterns of metabolites associated with carbohydrate, fat, and protein metabolism and stress

**Fig. 6:**
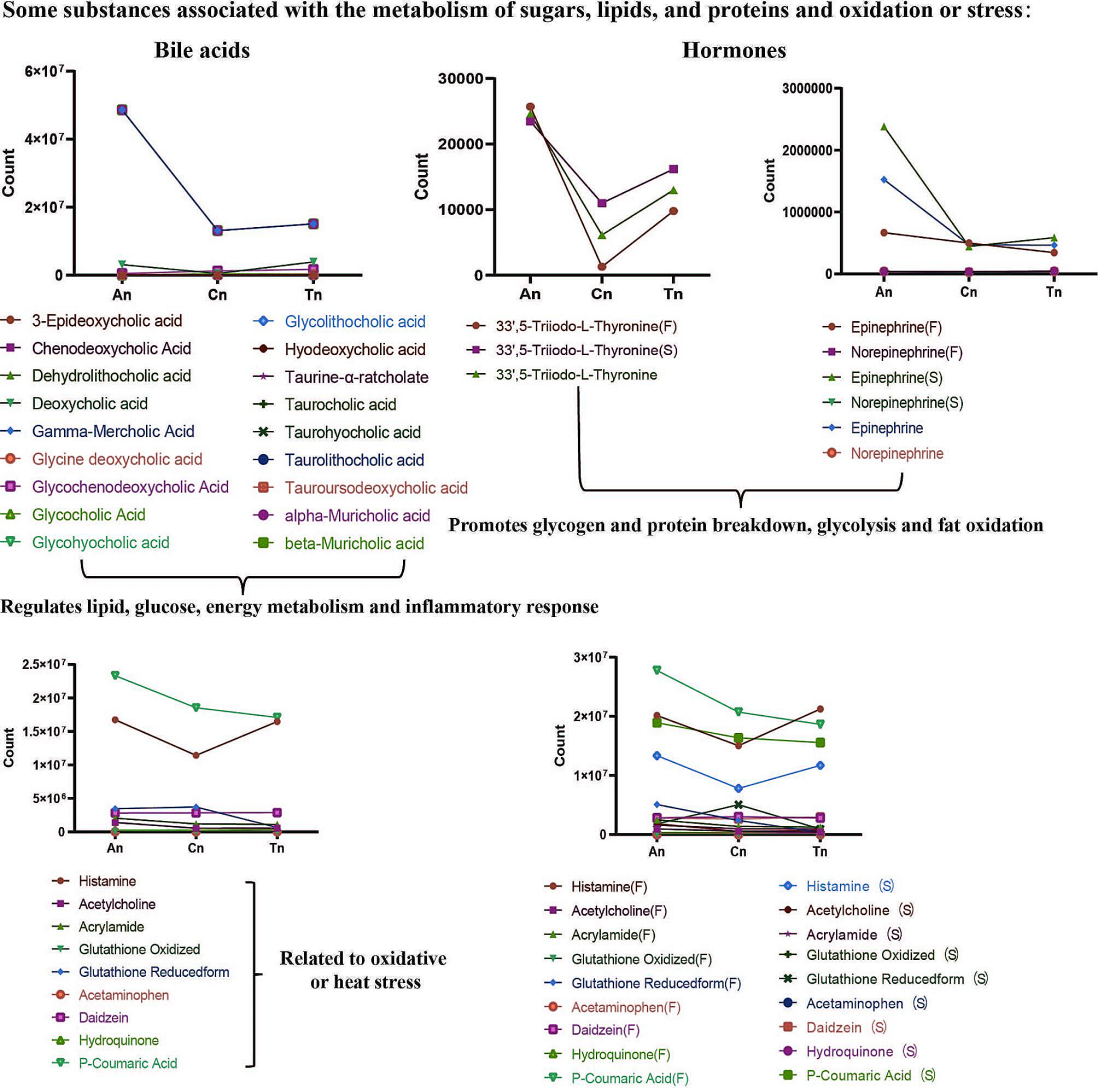
Expression trends of metabolites related to sugar, lipid, and protein metabolism and stress regulation

From An to Tn, we detected a total of 18 bile acids in the skin tissues of cashmere goats (Fig. 6 and Table S15). The downregulated expression trends of beta-muricholic acid (*P* < 0.01, VIP: 1.78, and AUC: 1 in An_F vs. An_S), gamma-mercholic acid (*P* < 0.01, VIP: 1.78, and AUC: 1 in An_F vs. An_S), and alpha-muricholic acid (*P* < 0.01, VIP: 1.78, and AUC: 1 in An_F vs. An_S) were extremely significant under grazing from An to Cn, and the expression of these three bile acids tended to be stable and remained at a relatively low level from Cn to Tn. Furthermore, the changes in the expression of other bile acids remained steady, and their levels decreased from An to Tn.

The hormones detected in the skin tissues were mainly alcoholic ketones, thyroid hormones, epinephrine, and norepinephrine (Fig. 6 and Table S15). Further analysis revealed that the pattern of 3,3’,5-triiodo-L-thyronine (*P* < 0.01, VIP: 1.68, and AUC: 0.92 in Cn vs. An; *P* < 0.01, VIP: 1.29, and AUC: 0.83 in Tn vs. An) expression was downregulated and then upregulated, with the F group showing a stronger trend than the S group. In contrast, the expression of epinephrine (*P* < 0.01, VIP: 1.26, and AUC: 0.90 in An vs. Cn; *P* < 0.01, VIP: 1.19, and AUC: 0.90 in An vs. Tn) significantly decreased from An to Cn and slightly increased from Cn to Tn, while the expression of norepinephrine decreased from An to Tn. Notably, during An, the expression of epinephrine (*P* < 0.01, VIP: 1.62, and AUC: 1) in skin tissue was significantly greater under grazing than under barn feeding.

Finally, we concentrated on the expression of metabolites linked to oxidative and heat stress (Fig. 6 and Table S15). The results showed that acetylcholine (*P* < 0.01, VIP: 1.49, and AUC: 0.99 in An vs. Cn; *P* < 0.01, VIP: 1.37, and AUC: 0.95 in Tn vs. An), acrylamide (*P* < 0.01, VIP: 1.40, and AUC: 0.85 in Cn vs. An; *P* < 0.01, VIP: 1.40, and AUC: 0.89 in Tn vs. An), glutathione reduced form (*P* < 0.05, VIP: 1.13, and AUC: 0.875 in Tn vs. An), and P-coumaric acid were downregulated from An to Tn. Although the overall trend of glutathione was downregulated, glutathione was upregulated and then downregulated in Group S (Fig. 6 and Table S15). Meanwhile, the expression of histamine was downregulated and then upregulated from An to Tn. In addition, the expression of histamine, acrylamide, acetaminophen (*P* < 0.05, VIP: 1.48, and AUC: 0.94 in An_F vs. An_S), glutathione oxidized, hydroquinone (*P* < 0.01, VIP: 1.83, and AUC: 1 in An_F vs. An_S; *P* < 0.01, VIP: 1.51, and AUC: 0.94 in Cn_F vs. Cn_S; *P* < 0.01, VIP: 1.57, and AUC: 0.94 in Tn_F vs. Tn_S), and P-coumaric acid in Group F was greater than that in Group S in all 3 stages. However, the expression of acetylcholine (*P* < 0.01, VIP: 1.62, and AUC: 0.94 in Tn_F vs. Tn_S) in An and Tn, glutathione reductase in Cn and Tn, and daidzein (*P* < 0.01, VIP: 1.54, and AUC: 0.89 in Cn_F vs. Cn_S) in Tn in Group F was lower than that in Group S.

### Expression pattern of prostaglandins in skin tissues

**Fig. 7:**
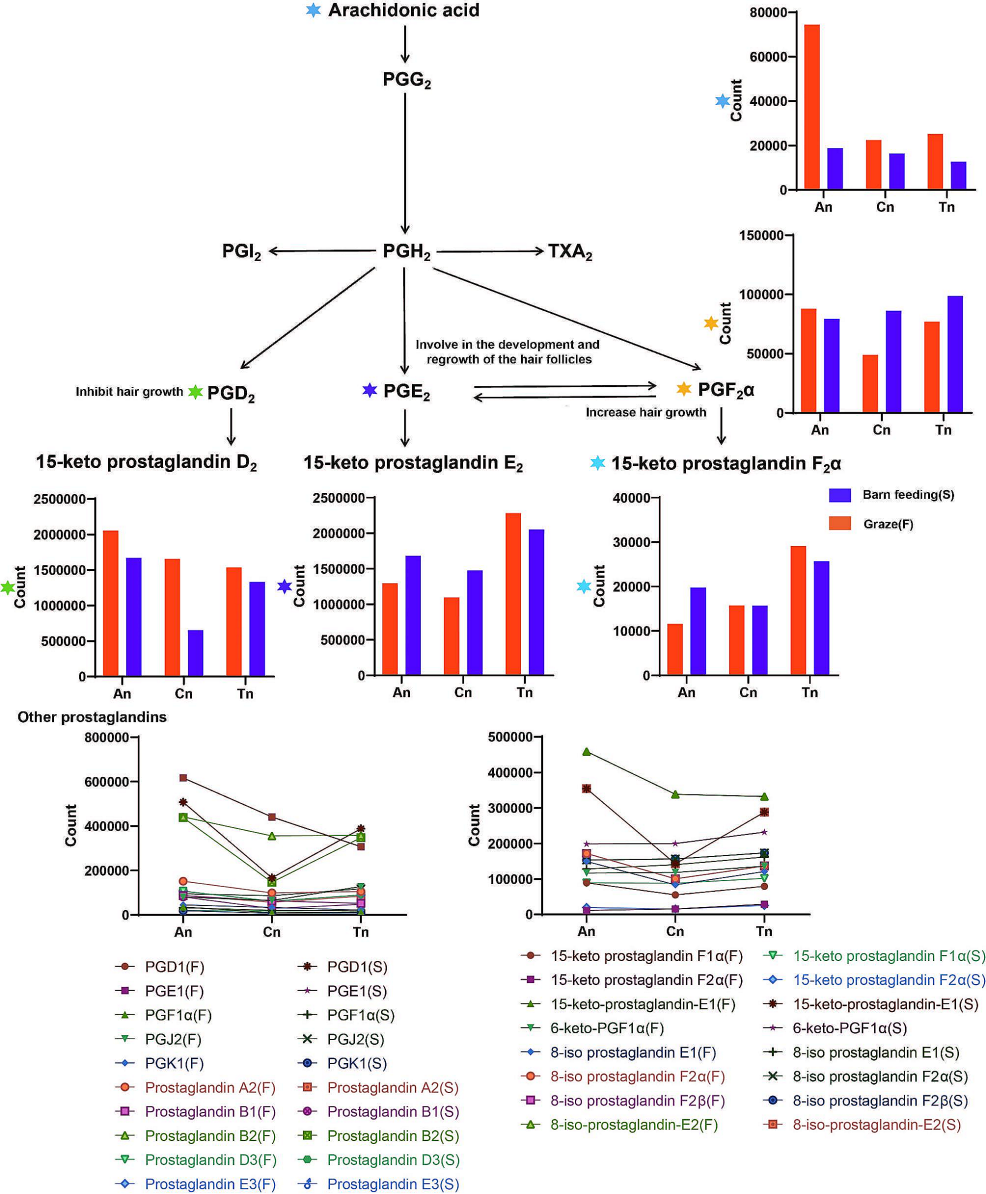
The metabolic pathway of prostaglandins and expression patterns of prostaglandins in skin tissue. Some metabolites and their corresponding expression columns are labeled with colored six-point stars

In the 36 metabolome samples, 22 prostaglandins and their metabolites were found (Fig. 7 and Table S16). Prostaglandins are biosynthesized from AA. Compared with those in the S group, the expression patterns of AA (*P* < 0.05, VIP: 1.21, and AUC: 0.73 in Cn vs. An; *P* < 0.05, VIP: 1.16, and AUC: 0.72 in Tn vs. An; *P* < 0.01, VIP: 1.80, and AUC: 1 in An_F vs. An_S; *P* < 0.01, VIP: 1.33, and AUC: 0.94 in Cn_F vs. Cn_S; *P* < 0.01, VIP: 1.75, and AUC: 1 in Tn_F vs. Tn_S) were significantly greater in the F group. Overall, the expression of PGD_2_, prostaglandin E_2_ (PGE_2_), and 15-keto prostaglandin F_2_α decreased and then increased from An to Tn. However, the expression of PGD_2_ and 15-keto prostaglandin F_2_α was downregulated and upregulated, respectively, from An to Tn. In addition, the expression of PGD_2_ (*P* < 0.01, VIP: 1.66, and AUC: 1) in Group F was significantly greater than that in Group S during Cn.

## Discussion

During the hair follicle cycle, hair follicle tissue eventually undergoes apoptosis from An to Tn. This is a tightly controlled process in which genes or new proteins are no longer produced or synthesized, and cell metabolism ceases. Several significant genes and signaling pathways are involved. However, this process requires not only the regulation of genes but also the participation of metabolites such as sugars, lipids, and proteins. The role of most metabolites in the hair follicle cycle has long been unknown. Furthermore, the form and expression of metabolites are frequently altered by a number of external stimuli. The impact of environmental variables on hair follicle cycle-related metabolites is also unclear. To the best of our knowledge, this work is the first to describe the temporal expression profiles of metabolites in skin tissues from An to Tn in Jiangnan cashmere goats, filling a gap in metabolite change patterns in hair follicle cycle investigations.

### The relationship between metabolites and the hair follicle cycle

#### The relationship between amino acids, their metabolites and hair follicle cycle

Keratin is the most abundant protein in animal hair, and cashmere keratin is mostly composed of the following amino acids: L-arginine, L-asparagine, L-proline, L-glycine, L-alanine, L-valine, L-tyrosine, L-phenylalanine, L-histidine, and L-threonine. Hence, small peptides (small peptides are oligopeptides composed of 2–3 amino acids) and amino acids act as substrates for keratin production in skin tissue. In our study, we consider that a decreasing trend in the expression of above metabolites (Fig. 2) is associated with the degeneration of hair follicles from An to Tn. In addition, Tang et al. identified a small peptide (tiger17, c[WCKPKPKPRCH-NH2]) in humans that was associated with skin wound healing [41], and Hirai et al. reported that the structurally optimized small peptide pep7 derived from epimorphin was effective in inducing hair follicle growth [42]. Cruz et al. showed that hydrophobic interactions and disulfide bond formation between the small peptides and human hair keratin were the main drivers of cosmetic peptide interactions, i.e., those aimed at improving human hair [43]. Above previous studies also confirmed a relationship between small peptides and hair follicle or skin tissue development.

In a subsequent analysis, we identified two amino acids, L-valine and L-tryptophan, as DEMs common to both An vs. Cn and An vs. Tn, both of which exhibited downregulated in expression from An to Tn. They were enriched in several pathways, including ABC transporters, axon regeneration, and protein digestion and absorption. Previous studies have shown that ABC transporters use ATP binding and hydrolysis to drive the transmembrane transport of various substrates [44–46]. ABC transporters bring nutrients and other molecules into the cell or pump toxins, drugs, and lipids out of the cell. In summary, we also consider that amino acids and their derivatives regulate the hair follicle cycle by participating in ABC transporters.

#### The relationship between nucleotides, their metabolites and the hair follicle cycle

Among many factors, genetic information determines the biosynthesis of keratin. Genetic information is conveyed from DNA to RNA, after which amino acids are progressively arranged and linked together to synthesize proteins according to the laws of the genetic code, and the genetic information is expressed in the order of the amino acids in such proteins. A nucleotide and its metabolites are directly involved in DNA or RNA synthesis, material or energy storage, and transport in the preceding processes. The downregulated expression of some purines and pyrimidines in our study may imply decreased tissue metabolism, which may also be connected with hair follicle tissue deterioration and apoptosis (Fig. 5).

According to the DEM analysis, 2’-deoxyadenosine and 2’-deoxyuridine were enriched in the ABC transporters, purine metabolism, or pyrimidine metabolism in the An vs. Cn and An vs. Tn. Guanosine-5’-monophosphate, a common DEM of the An vs. Cn, An vs. Tn, and Tn vs. Cn, was enriched in the cGMP-PKG signaling pathway and purine metabolism. Previous studies have shown that the cGMP/PKG signaling pathway play an important role in preventing the activation of the pro-apoptotic pathway and promoting neuronal cell survival [47]. Therefore, we further consider that nucleotides and their metabolites not only act as substrates for the biosynthesis of DNA and RNA, but also affect cellular energy metabolism and material transport in skin tissues by participating in ABC transporters and cGMP-PKG signaling pathway, thus regulating the degradation of hair follicles.

#### The relationship between lipids and the hair follicle cycle

In addition to amino acids, nucleotides, and their metabolites, lipids also play an irreplaceable role in the physiological and biochemical activities of animals. In this study, the GPs were detected in goat skin mainly included LPEs and LPCs. LPCs are intermediate product of PCs metabolism, and LPCs undergoe an esterification reaction to form PCs. PC is one of the most important phospholipids in eukaryotic cells, accounting for approximately 50% of the total cellular phospholipids. It plays a very important role in the structural stability of the cell membrane and intracellular signaling. Previous studies have shown that disturbances in the balance of PC metabolism lead to cell cycle arrest and even apoptosis [48]. In this study, we found that the expression of most LPCs (e.g., LPC(0:0/16:0), LPC(0:0/18:0), LPC(12:0/0:0), LPC(15:0/0:0), etc.) in An was higher than that in Cn and Tn (Fig. 2), which may be related to more active hair follicle stem cell development or division in An skin tissues.

In addition, numerous studies have shown that carnitines contribute to hair development in animals. For example, Andrieu-Abadie et al. [49] and Vescovo et al. [50] found that L-carnitine prevents apoptosis in cardiomyocytes and skeletal muscle cells, respectively. L-carnitine-L-tartrate has also been shown to promote human hair growth [51]. Brotzu et al. found that liposome formulations containing propionyl-L-carnitine could prevent and treat hair loss [52]. In our study, the results showed a decreasing trend in the expression of some carnitines (e.g., carnitine C18:2, carnitine C9:1, carnitine C17:1:DC, carnitine C16:1, carnitine C2:0, carnitine C5:0, carnitine C20:1, carnitine C22:2, etc.), and carnitine C18:2 was the common DEM among the An vs. Cn, Tn vs. Cn, and An vs. Tn, and carnitine C9:1 was the DEM in the An vs. Cn and An vs. Tn. Based on the results of previous studies, we speculate that the downregulated expression of these substances may be associated with the degeneration of hair follicle tissue or cell apoptosis.

#### The relationship between prostaglandins and the hair follicle cycle

Hair growth is regulated by several physiological factors, such as hormones, growth factors, cytokines, and adhesion molecules. In addition, prostaglandins, one of the cyclooxygenase metabolites of AA, are known to be associated with hair growth. For example, aspirin analogs (cyclooxygenase inhibitors) have been reported to induce hair loss in humans, while topical or systemic administration of PGE_2_ analogs protects mice from radiation-induced alopecia [53]. Furthermore, one of the therapeutic effects of the hair growth stimulant minoxidil is thought to be due to its stimulation of PGE_2_ synthesis, which leads to the induction and prolongation of the growth primordium phase of normal hair growth [54]. Moreover, Purba et al. demonstrated a possible link between PGD_2_ and disrupted proliferation kinetics in epithelial stem cells [55]. Garza et al. showed that prostaglandin D_2_ inhibits hair growth and is elevated during baldness in men with androgenetic alopecia [56]. In this study, we found that arachidonic acid and PGE_2_ tended to be downregulated from An to Cn, which may also be related to hair follicle degeneration (Fig. 7). However, the expression of PGE_2_ in skin tissues was higher in An than in other stages, which is contrary to the results of previous studies and deserves further investigation.

#### Potential effects of external factors on the hair follicle cycle and hair growth

In Xinjiang, the boundary between seasons is very clear: the summer has short, hot spells and long light hours, and the exact opposite occurs in the winter. This complex and vast geographical area has resulted in the scattered distribution of the Jiangnan cashmere goat population, and different regions have developed different cashmere goat breeding models based on their own conditions. Jiangnan cashmere goats are grazed in Aksu (primarily mountains, desert, and Gobi), and their major food source is plants cultivated outdoors. In contrast, Jiangnan cashmere goats in Qitai are mainly fed in barns, and their diet is mainly composed of silage, wheat grass, alfalfa, and mixed feed.

#### Light regulates hair follicle development by promoting the biosynthesis of thyroid hormone and vitamin D3

**Fig. 8:**
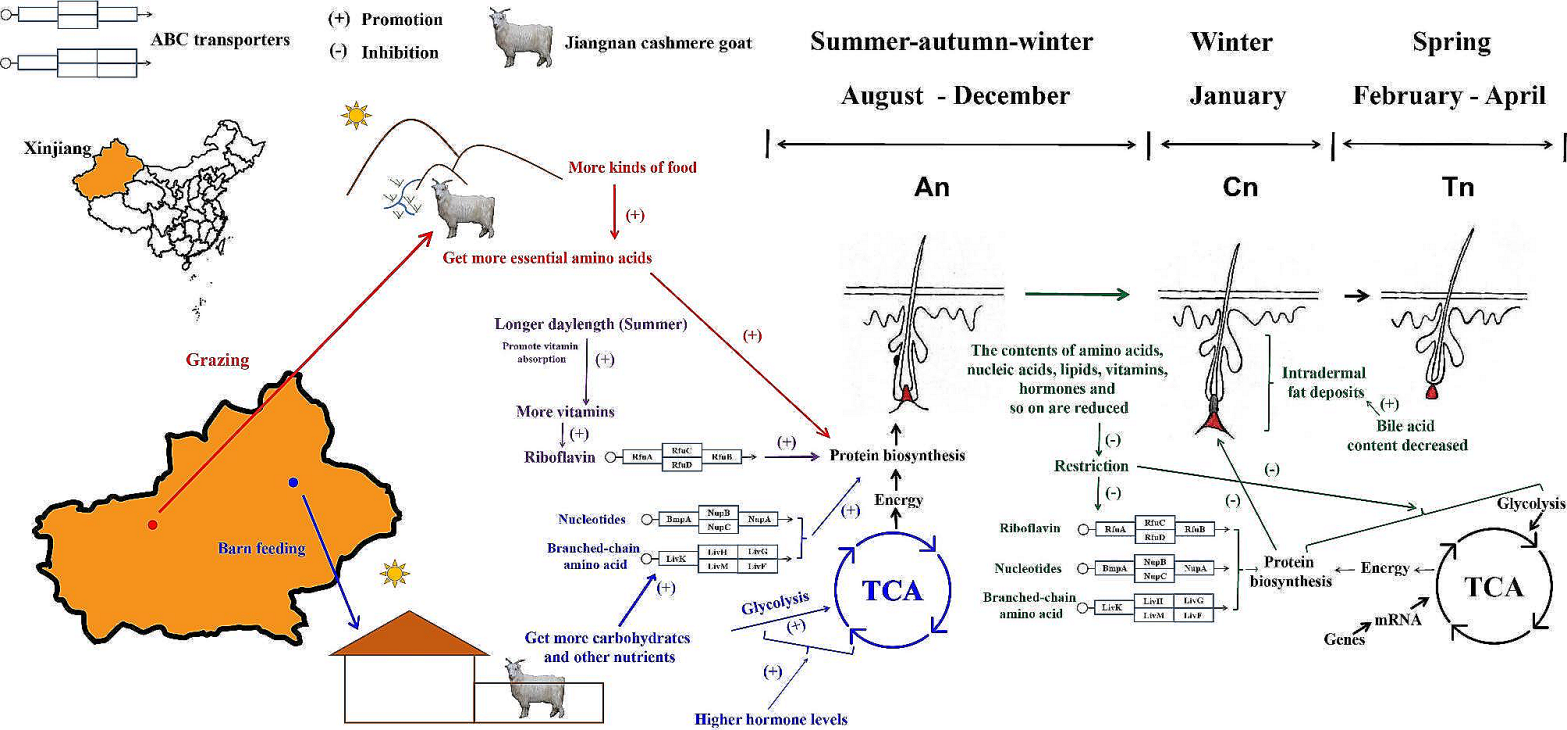
The potential role of metabolites in hair follicle cycles. We introduce in detail the distribution of the Jiangnan cashmere goat, the feeding models of the Jiangnan cashmere goat in Aksu and Qitai, the seasons of the three stages (An, Cn, and Tn), and the morphology of the hair follicles in these three stages. The map in Fig. 8 was drawn using map software in the R package

Light is an important factor affecting the production performance of livestock and poultry. Suitable light promotes milk, egg, and wool production in livestock or poultry, while short or weak light is beneficial for the deposition of adipose tissue. Research by Morris showed that intentionally and gradually limiting the amount of light hours can slow wool growth, while gradually increasing the length of light can accelerate it [57]. It is widely assumed that light stimulates the release of thyroid hormones in animals by boosting the secretion of thyroid-stimulating hormone. Thyroid hormones affect cell metabolism and development in animals, boosting nervous system excitability, and enhanced thyroid hormone synthesis promotes hair follicle development. Numerous studies have demonstrated that thyroxine, whether taken orally, injected, or buried, increases the growth of wool [58–60]. In this study, the expression level of 3,3’,5-triiodo-L-thyronine in the F group was higher than that in the S group at An, which could be attributed to cashmere goats being exposed to light for a longer period of time when grazing. Moreover, the expression levels of 3,3’,5-triiodo-L-thyronine and L-thyroxine decreased from An to Cn and subsequently increased from Cn to Tn, which may be related to seasonal changes in light hours (from summer to winter and back to spring) (Fig. 8). In summary, we assume that with the change in season, the decrease in light hours suppresses the secretion of thyroid hormones in cashmere goats, further leading to the degeneration of hair follicles. Melatonin also affects the hair follicle cycle in animals. Previous studies have shown that melatonin treatment prolongs the hair follicle cycle. The wool weight and fiber diameter of goats administered subcutaneous melatonin were significantly greater than those in the oral melatonin and control groups [61]. However, melatonin was not detected in the skin tissues of cashmere goats in all three stages in this study.

Meanwhile, the expression pattern of vitamin D3 was similar to that of thyroxine in this study. Vitamin D produces many biological effects by binding to the vitamin D receptor (VDR), including the regulation of calcium and phosphorus metabolism to maintain skeletal and immune system stability and cell development. Recent studies have shown that vitamin D and its receptors play a vital role in hair development. Vitamin D receptor deficiency disrupts the Wnt signaling pathway in hair follicle stem cells, resulting in aberrant hair follicle stem cell proliferation and differentiation and hair loss in the animal, eventually [62]. Because photosynthesis in the skin (generated by UV irradiation) and dietary supplements (to a lesser extent) are the main sources of vitamin D, the skin tissues of cashmere goats are prone to synthesizing higher levels of vitamin D3 during the summer (Fig. 8). Our findings suggest that greater light exposure may impact the metabolism of basal chemicals in cashmere goats by boosting the production of thyroid hormones, which in turn regulate hair follicle growth. Moreover, light may influence the hair follicle cycle by influencing vitamin D3 levels in skin tissues.

#### Temperature is one of the factors that affects the hair follicle cycle

Various physiological processes and cellular metabolism in animals are carried out within a certain temperature range. Within a particular range, physiological and biochemical reactions in animals increase with increasing temperature, thereby accelerating growth and development, and they are retarded with decreasing temperature, which delays growth and development. Adipose tissue plays an indispensable role in responding to environmental changes since it does not transport heat easily and serve as an excellent insulating layer. Qu and Ajuwon found that high temperatures increased the expressions of triglycerides, phosphatidylinositol, phosphatidylserine, saturated fatty acids, monounsaturated fatty acids, and carnitines in adipocytes while decreasing the accumulation of monoacylglycerols, phosphatidylcholine, phosphatidylethanolamine, and phosphatidylglycerol in pigs [63]. Based on Qu and Ajuwon’s study, ambient temperature regulates lipid metabolism in animals, high temperatures block fatty acid oxidation and boost lipogenic pathways. Furthermore, rapid downward hair follicle growth is connected with thickening of the dermal adipose layer, and the expansion of the adipose layer promotes hair follicle growth. Schmidt and Horsley’s research revealed that intradermal adipocytes regenerate quicker than other adipose tissue cells, and match the hair follicle cycle [64]. In summary, ambient temperature may affect the hair follicle cycle by modulating fat metabolism in the skin tissue. The expression of most fatty acids (e.g., FFA(22:7), FFA(16:1), FFA(12:0), FFA(18:2), and FFA(20:0)) in goat skin tissue was greater in An than in Cn (Fig. 2B), which is consistent with earlier findings and may be related to intradermal fat accumulation. Moreover, from An to Cn, the season in Xinjiang progressively changes from summer to winter, and the temperature decreases (Fig. 8). Hence, we believe that a decrease in temperature promotes adipose deposition in skin tissue, thereby regulating the hair follicle cycle.

Bile acids are critical to the oxidative degradation of lipids. Bile acids not only aid in fat digestion and absorption in the body but also regulate substance metabolism (lipid, glucose, or energy) and inflammatory responses via farnesol X receptor- and transmembrane G protein-coupled receptor 5-dependent mechanisms [27, 65]. The decrease in bile acid levels in skin tissue from An to Cn (Fig. 5) may be related to intradermal fat deposition, which in turn promotes the formation of cashmere goat hair follicles.

#### The effect of different feeding modes on hair follicle development

Some important metabolites involved in the formation of animal tissues can only be obtained through feeding during growth and development; essential amino acids are among these important metabolites, and some of them (Phe, Met, Lys, Tyr, Thr, Val, Iso, and Leu) (Fig. 2B) are mentioned above as important components of goat cashmere keratin. Furthermore, our findings suggest that consuming a wide variety of food under grazing may make it easier for cashmere goats to obtain more necessary amino acids, which is favorable for cashmere growth. Cashmere goats can acquire more carbohydrates from silage in barn feed than they can from grazing, which can supply energy for animal cell metabolism via glycolysis and the TCA cycle (Fig. 8). Glycans can also be used to manufacture lipids and nonessential amino acids via gluconeogenesis and other processes, supplying raw materials for the creation of animal tissues and structures and benefiting goat cashmere growth.

B vitamins consist of eight water-soluble vitamins (thiamine (B1), riboflavin (B2), niacin (B3), pantothenic acid (B5), vitamin B6, biotin (B7), folic acid, and vitamin B12), which contribute to cellular metabolism. With the exception of biotin, a balanced diet may provide the required daily consumption of these vitamins. Among them, riboflavin, biotin, folic acid, and vitamin B12 deficiencies are associated with hair loss [66]. We consider that the downregulated expression trend of riboflavin may be related to the hair follicle cycle and that the rich variety of food under grazing is also conducive to the consumption of more riboflavin by cashmere goats. Moreover, the KEGG enrichment analysis revealed that riboflavin was enriched in ABC transporters, implying that riboflavin may regulate the hair follicle cycle by engaging in ABC transporters to influence substance transport.

### The side effects of stress

It is also worth noting that long-term grazing or barn feeding might cause animal stress. Among alcohols and amines, phenolic acids, amino acids and their metabolites, some metabolites, such as histamine [67], acrylamide [68], glutathione [69], acetaminophen [70], and daidzein [71], are associated with coping with stress and inflammation. In An, these metabolites were expressed at higher levels in cashmere goat skin tissue, and the expression levels of these metabolites in the F group were greater than those in the S group (Fig. 6). This suggests that grazing causes greater stress in cashmere goats, resulting in more carbohydrates being anaerobically converted to lactate in the skin of cashmere goats, decreasing carbohydrate utilization efficiency. Randhawa et al. found that increased light intensity enhanced the anaerobic metabolic pathways of glucose and the aerobic metabolic pathways of lipids in skin tissues, as evidenced by glucose, lactate, 3-phosphoglycerate carnitine, and glycerol, while glutathione content decreased [72], which is similar to our findings and may result in premature aging of the skin. Although grazing supports cashmere growth to some extent, the harm caused by overgrazing of cashmere goats cannot be ignored.

## Conclusions

This work demonstrates for the first time the pattern of metabolite expression in cashmere goat skin tissue from anagen to telogen. The expression trends and differences of some metabolites (sugars, amino acids, lipids, nucleotides, vitamins, and hormones) reflect the potential relationships between external factors (light, temperature, and feeding modes), some important metabolites, and the hair follicle cycle. Among the above DEMs, 2’-deoxyadenosine, L-valine, 2’-deoxyuridine, riboflavin, cytidine, deoxyguanosine, L-tryptophan, and guanosine-5’-monophosphate may regulate the hair follicle cycle by participating in ABC transporters.

## Materials and methods

### Animals

Twelve 24-month-old female Jiangnan cashmere goats were chosen for this study from the Xinjiang Aksu Baihutai Cashmere Goat Breeding Center (Aksu) (*n* = 6) and the Xinjiang Kechuang Livestock Breeding Center (Qitai) (*n* = 6). The twelve goats were healthy, and their developmental and physiological statuses were similar and good. In the Xinjiang Aksu Baihutai Cashmere Goat Breeding Center, cashmere goats are raised by grazing, and the food source of the goats is a variety of plants in the wild. Conversely, the cashmere goats raised at the Xinjiang Kechuang livestock breeding center are barn-fed, and they are fed mixed feed consisting of 76% silage, 4% wheatgrass, 10% alfalfa, and 10% concentrate. Concentrate feed (product standard No. Q/TC010-2023) was obtained from Xinjiang Tiankang Feed Co., Ltd.

### Sample collection and treatment

A previous study revealed that the general development of hair follicles in Jiangnan cashmere goats raised by the Xinjiang Aksu Baihutai Cashmere Goat Breeding Center and Xinjiang Kechuang Livestock Breeding Center was the same [73]. In September, January, and March, most of the hair follicles of the 12 Jiangnan cashmere goats were in An, Cn, and Tn, respectively. Hence, 1 cm^2^ of intact skin tissue (including the epidermis, dermis and subcutaneous tissue) was collected from the posterior border of the left scapula of these 12 cashmere goats in September (An) and January (Cn) of the same year and in March (Tn) of the following year. A total of 36 skin tissue samples (An: *n* = 12, Cn: *n* = 12 and Tn: *n* = 12) were eventually collected. The skin tissues were washed with PBS (Solarbio, Beijing) before being frozen in liquid nitrogen. We bandaged and treated the wounds of the experimental animals to ensure that they were not infected with bacteria, and all of the experimental animals were still healthy after the sampling was finished. Finally, Biomarker Technologies Co. Ltd. (Beijing, China) performed the metabolite extraction, detection, and quantification. The skin metabolites were then extracted using the procedures described by Dunn et al. [74] and Want et al. [75]. The main extraction process is as follows:

1. The skin tissue was freeze-dried under vacuum in a Scientz-100 F freeze-dryer (SCIENTZ, China).
2. The skin tissue was ground (30 Hz, 1.5 min) to a powder using an MM 400 grinder (Retsch, Germany).
3. One hundred milligrams of powder was weighed and dissolved in 1.2 mL of 70% methanol (Merck, Germany).
4. The sample was swirled every 30 min for 30 s each time for a total of 6 vortices, after which the sample was placed in a 4 °C refrigerator overnight.
5. The sample was removed from the refrigerator and centrifuged (speed: 12,000 rpm, 10 min).
6. After centrifugation, the supernatant was collected, and the sample was filtered through a microporous filter membrane (0.22 μm pore size) and stored in a sample vial for UPLC‒MS/MS analysis.

### Metabolite detection and quantification

In this study, ultra-performance liquid chromatography (UPLC) (SHIMADZU Nexera X2, https://www.shimadzu.com.cn/) and tandem mass spectrometry (MS/MS) (Applied Biosystems QTRAP, http://www.appliedbiosystems.com.cn/) were used for metabolite detection in 36 skin tissue samples. The liquid phase settings and mass spectrometry conditions were the same as those described by Wang et al. [76] and Garcia et al. [77].

The UPLC conditions mainly include the following:

1. The chromatographic column used was an Agilent (Germany) SB-C18 column (1.8 μm, 2.1 mm*100 mm).
2. For the mobile phase, phase A was ultrapure water (0.1% formic acid (Merck, Germany)), and phase B was acetonitrile (Merck, Germany) (0.1% formic acid).
3. Elution gradient: at 0.00 min, the proportion of phase B was 5%; within 9.00 min, the proportion of phase B linearly increased to 95% and was maintained at 95% per minute; at 10.00–11.10 min, the proportion of phase B was reduced to 5% and was balanced at 5% to 14 min.
4. Flow rate: 0.35 ml/min; column temperature: 40 °C; sample size: 4 µl.

The MS conditions mainly included:

LIT and triple quadrupole (QQQ) scanning were performed on a triple quadrupole linear ion TRAP mass spectrometer (Q TRAP) and an AB6500 Q TRAP UPLC/MS/MS system equipped with an ESI Turbo ion spray interface. The ESI source operating parameters were as follows: ion source, turbo spray; source temperature, 550 °C; ion spray voltage, 5500 V (positive ion mode)/-4500 V (negative ion mode). The ion source gas I, gas II, and curtain gas were set to 50, 60, and 25.0 psi, respectively, and the collision-induced ionization parameter was set to high. The instrument was tuned and calibrated with 10 and 100 µmol/L polypropylene glycol solutions (Merck, Germany) in the QQQ and LIT modes, respectively. The instrument was tuned and calibrated with 10 and 100 µmol/L polypropylene glycol solutions in the QQQ and LIT modes, respectively. Based on further DP and CE optimization, the DP and CE of each MRM ion pair were determined. A specific set of MRM ion pairs was monitored at each period based on the metabolite elution during each period.

On the basis of a local metabolic database, 36 samples were subjected to qualitative and quantitative mass spectrometric analysis of metabolites. Isotopic signals, repeated signals containing K^+^ ions, Na^+^ ions, and NH_4_^+^ ions, as well as repeated signals of fragment ions that are themselves other larger molecular weight substances, were removed during the analysis. Metabolite quantification was performed via multiple reaction monitoring analysis via triple quadrupole mass spectrometry. The characteristic ions of each substance were screened by QQQ, the signal intensity of the characteristic ions was obtained with a detector, the sample offline mass spectrometry file was opened with MultiQuant software, peak integration and calibration were performed, the peak area of each peak represented the relative content of the corresponding substance, and finally, all of the peak area integration data were exported and stored.

### Data processing and statistical analysis

First, PCA was performed on 36 skin metabolomes using scales and ggplot2 software in the R package based on the expression of all metabolites (parameter setting: scale: UV scaling). Then, the expression trend analysis of all metabolites from An to Tn was performed using the short time-series expression miner (STEM) software [78], clustering method: STEM clustering method. Correlation analysis between samples was performed using pheatmap software (scale: UV scaling) in the R package, and correlation heatmaps were generated.

The R package ropls [79] was used to execute orthogonal partial least squares-discriminant analysis (OPLS-DA) (parameter setting: number of cross-validation folds: 7, number of replacement tests: 200). The DEMs for different stages or different feeding models were screened from the variable importance in projection (VIP) of the OPLS-DA model in combination with *P*-value or difference multiplier (fold change) values from univariate analysis. Screening criteria of DEMs were fold change ≥ 1, VIP ≥ 1, and *P*-value < 0.05. VIP ≥ 1 is generally considered to be significantly expressed differently. The DEMs were annotated using the KEGG database. Finally, MetaboAnalystR [80], caTools, and pROC software in the R package were used to perform ROC analysis on each group of screened DEMs, and their AUC values were determined. ROC algorithm: Wilcoxon rank-sum test; log transformation: generalized log transformation. In ROC curve analysis, the AUC values for significantly DEMs (VIP ≥ 1 and *P*-value < 0.05) in the six comparison groups were calculated. AUC values range from 1 (there is a threshold for perfect separation between classes) to 0.5 (two classes are statistically identical).

### Electronic supplementary material

Below is the link to the electronic supplementary material.

Supplementary Material 1

## Data Availability

Data is contained within the article or supplementary material.

## Abbreviations

DEMs: Differentially expressed metabolites
An: Anagen
Cn: Catagen
Tn: Telogen
AA: Arachidonic acid
PGD_2_: Prostaglandin D_2_
PGE_2_: Prostaglandin E_2_
ROC: Receiver operating characteristic
VIP: Importance in projection
AUC: Area under the curve
TCA: Tricarboxylic acid cycle
UPLC: Ultra-performance liquid chromatography
MS: Mass spectrometry
QQQ: Triple quadruple
OPLS-DA: Rthogonal partial least squares discriminant analysis
GP: Glyceryl phosphatide
LPC: Lyso-phosphatidylcholine
LPE: Lysophosphatidyl ethanolamine
LPA: Lysophosphatidic acid
PI: Phosphatidyl inositol
PE: Phosphatidyl ethanolamine
PC: Phosphatidylcholine
GL: Glycerolipid
TG: Triglyceride
FA: Fatty acyl
FFA: Free fatty acid
OPLS-DA: Rthogonal partial least squares discriminant analysis
Phe: Phenylalanine
Met: Methionine
Lys: Lysine
Tyr: Tyrosine
Thr: Threonine
Val: Valine
Iso: Isoleucine
Leu: Leucine
Cys: Cysteine
Ala: Alanine
Glu: Glutamic acid
Gly: Glycine
Cys: Cystine
Cys: Cystine
Pro: Proline
Try: Tryptophan
Try: Tryptophan
Asp: Aspartic acid
His: Histidine
